# Influence of Harvest Aid Herbicides on Seed Germination, Seedling Vigor and Milling Quality Traits of Red Lentil (*Lens culinaris* L.)

**DOI:** 10.3389/fpls.2017.00311

**Published:** 2017-03-14

**Authors:** Maya Subedi, Christian J. Willenborg, Albert Vandenberg

**Affiliations:** Department of Plant Sciences, University of SaskatchewanSaskatoon, SK, Canada

**Keywords:** dehulling efficiency, football recovery, milling recovery, pre-harvest aid, desiccant, lentil

## Abstract

Most red lentil produced worldwide is consumed in dehulled form, and post-harvest milling and splitting qualities are major concerns in the secondary processing industry. Lentil producers in northern temperate regions usually apply pre-harvest desiccants as harvest aids to accelerate the lentil crop drying process and facilitate harvesting operations. This paper reports on field studies conducted at Scott and Saskatoon, Saskatchewan, Canada in the 2012 and 2013 cropping seasons to evaluate whether herbicides applied as harvest aids alone or tank mixed with glyphosate affect seed germination, seedling vigor, milling, and splitting qualities. The site-year by desiccant treatment interaction for seed germination, vigor, and milling recovery yields was significant. Glyphosate applied alone or as tank mix with other herbicides (except diquat) reduced seed germination and seedling vigor at Saskatoon and Scott in 2012 only. Pyraflufen-ethyl (20 g ai ha^−1^) applied with glyphosate as well as saflufenacil (36 g ai ha^−1^) decreased dehulling efficiency, while saflufenacil and/or glufosinate with glyphosate reduced milling recovery and football recovery, although these effects were inconsistent. Application of diquat alone or in combination with glyphosate exhibited more consistent dehulling efficiency gains and increases in milling recovery yield. Significant but negative associations were observed between glyphosate residue in seeds and seed germination (*r* = −0.84, *p* < 0.001), seed vigor (*r* = −0.62, *p* < 0.001), dehulling efficiency (*r* = −0.55, *p* < 0.001), and milling recovery (*r* = −0.62, *p* < 0.001). These results indicate application of diquat alone or in combination with glyphosate may be a preferred option for lentil growers to improve milling recovery yield.

## Introduction

Lentil (*Lens culinaris* Medik.) is a valuable grain legume crop that is a good source of dietary protein, fiber, complex carbohydrates, and minerals (Jood et al., 1998; Xu and Chang, 2009; DellaValle et al., 2013). The Western Canadian Prairies is the world's major lentil producing and exporting region, with a current production area of 2.3 million ha (Statistics Canada, 2016). The main destinations for red lentil are India, Turkey, the United Arab Emirates, and the European Union (Statistics Canada, 2016). About 90% of red lentils are consumed in dehulled form after removal of the seed coat through an abrasive dehulling process that improves the taste of cooked lentil by removing anti-nutritional factors, such as polyphenols and tannins, which are mostly retained in the seed coat fraction (Singh and Singh, 1992; Wang, 2008; DellaValle et al., 2013). Efficient loosening of the seed coat during dehulling process of red lentil is vital for the milling industry. The market value of red lentil largely depends on milling quality, which in turn depends on genetics but also on agronomic practices, such as the application of pre-harvest desiccants and growing environment (Ramakrishnaiah and Kurien, 1983; Wang, 2008).

Lentils harvested under low humidity and high temperature conditions in Australia, Mediterranean and sub-tropical savannah climates are more efficiently dehulled than those harvested in a temperate climate (Brand, 2008). Northern temperate prairie environments experience much different climatic conditions compared to global competitors who tend to harvest during hot dry conditions. Indeed, red lentils produced in northern temperate prairie regions generally have a higher moisture content at harvest and different physical characteristics than those grown in Mediterranean and sub-tropical savannahs. The climate, genetic base, and agronomic practices for red lentil grown in northern temperate climates are unique, and may result in poor milling recovery compared to lentil produced and milled in dry environments (Vandenberg, 2009).

In Western Canada, the environmental variation within fields and the indeterminate nature of crop growth means that lentil growers usually apply pre-harvest desiccants to optimize harvest conditions on the variable landscape. Lentils are considered sufficiently mature for desiccation with harvesting aids when 80% of the pods in the lower third of the canopy have turned from green to yellow or brown (Saskatchewan Pulse Growers, 2015). Desiccant chemistry and application timing are crucial as they may cause loss of yield and quality (Bennett and Shaw, 2000a; Wilson and Smith, 2002; Zhang et al., 2016). In Western Canada, the herbicides registered as harvest aids for lentil and other pulses include diquat, glyphosate, saflufenacil, glufosinate, and flumioxazin (Risula, 2014). These desiccants have different modes of action and chemistry and, therefore, may differentially affect post-harvest seed quality.

Diquat is a quick acting contact herbicide that is traditionally used as a harvest aid for lentil. It rapidly and quickly dries plant tissues within few days of application and has no or low translocation in plants (Cobb and Reade, 2010; Zhang et al., 2016). However, pre-harvest application of diquat affects milling recovery yield and other post-harvest seed qualities in lentil when it is applied too early (Bruce, 2008). Glyphosate, a popular herbicide product, is used in lentil production to control late emerging annual weeds and acts as a desiccant. It is also used as a desiccant in common bean production in Canada (McNaughton et al., 2015). Seeds are a major photosynthate sink during maturation (Cakmak et al., 2009); glyphosate is translocated mainly through phloem and distributed throughout the plant, and seed germination and vigor may be reduced if seeds accumulate too much glyphosate residue (Clay and Griffin, 2000; Zhang et al., 2016). Glufosinate and saflufenacil are newly registered as desiccants for lentil crops (Risula, 2014). Saflufenacil is a weak acid herbicide used for broadleaf weed control in soybean, corn, and other crops. It moves both acropetally and basipetally in plants and affects symptoms similar to other contact herbicides (Soltani et al., 2010). Glufosinate can translocate within plants, but has rapid phytotoxicity that limits its mobility (Grossmann et al., 2010; Soltani et al., 2013). Pyraflufen-ethyl and flumioxazin are also potential harvest aids for lentil crops in Western Canada (Risula, 2014; Zhang et al., 2016) and are commonly used as desiccants in cotton (Griffin et al., 2010) and common bean (Soltani et al., 2013). Overall, minimal research has been conducted to assess the impact of the complete spectrum of harvest aids (e.g., diquat, pyraflufen, glufosinate ammonium, flumioxazin, and saflufenacil) alone or in combination with glyphosate with respect to their effects on seed biology and post-harvest processing of lentil. This study was conducted to evaluate the effect of contact herbicides applied alone or as tank mixtures with glyphosate as harvest aids on milling recovery yield, seed germination, seedling vigor, and other seed quality attributes of red lentil.

## Materials and methods

### Field experiments

Harvested seed samples of red lentil (cv. CDC Maxim) were obtained from field experiments conducted by Zhang et al. (2016) in two Saskatchewan locations in 2012 and 2013. These locations (Saskatoon, 52°36′ N, 108°84′ W, altitude 659.6 m; Scott, 52°09′ N, 106°33′ W, altitude 505 m) are located in the Dark Brown zone of Chernozemic soils (clay to sandy loam at Saskatoon and silt loam at Scott). The soil organic matter content and pH ranged from 2.4 to 4.5% and 7.5 to 7.9 at Saskatoon and 2.4 to 2.6% and 5.3 to 6.8 at Scott, respectively.

The experimental treatments (Table 1) were arranged in a randomized complete block design (RCBD) with four replicates. Each block consisted of 21 desiccants and an unsprayed control. The desiccants used in this study were pyraflufen-ethyl (10 and 20 g ai ha^−1^), glufosinate (300 and 600 g ai ha^−1^), flumioxazin (105 and 210 g ai ha^−1^), saflufenacil (36 and 50 g ai ha^−1^) and diquat (208 and 415 g ai ha^−1^), with each desiccant applied alone or in combination with glyphosate (900 g ae ha^−1^). The list of treatments is presented in Table 1. All desiccant treatments were applied with the recommended adjuvant, either Merge® (50% surfactant; 50% petroleum hydrocarbons solvent) or Agral 90®; (90% nonylphenoxy polyethoxy ethanol) with an air pressurized tractor mounted sprayer equipped with shielding (110–015 AirMix nozzles, 275 kpa, 45 cm spacing at Saskatoon and with a CO_2_ pressurized bicycle sprayer (110–003 AirMix nozzles, 276 kpa, 25 cm) at Scott. Both sprayers were calibrated to deliver 200 L ha^−1^ of spray solution. Prior to crop harvest aid applications, the seed moisture content was determined by picking and bulking a few seeds from two border plots to create a composite sample, which was then dried at 90°C for 24 h. Treatments were applied to the lentil crop at approximately 30% seed moisture content at the recommended stage (when lower seeds rattled and pods started turning brown, and middle pods were yellow to brown). The crops were harvested 21 days after desiccant application.

**Table 1:** **Desiccant (pre-harvest aids) treatments and their rate of application**.

**Desiccant /Herbicide**	**Rate (g a.i./a.e. ha^−1^)**	**Surfactants**
Untreated check		-
Glyphosate	900	-
Pyraflufen	10	Merge (1% v/v)
	20	Merge (1% v/v)
Pyraflufen+Glyphosate	10 + 900	Merge (1% v/v)
	20 + 900	Merge (1% v/v)
Glufosinate	300	-
	600	-
Glufosinate + Glyphosate	300 + 900	-
	600 + 900	-
Flumioxazin	105	Agral 90 (0.25% v/v)
	210	Agral 90 (0.25% v/v)
Flumioxazin+Glyphosate	105 + 900	Agral 90 (0.25% v/v)
	210 + 900	Agral 90 (0.25% v/v)
Saflufenacil	36	Merge (1% v/v)
	50	Merge (1% v/v)
Saflufenacil + Glyphosate	36 + 900	Merge (0.5% v/v)
	50 + 900	Merge (0.5% v/v)
Diquat	208	Agral (0.1%v/v)
	415	Agral (0.1%v/v)
Diquat+Glyphosate	208 + 900	Agral (0.1%v/v)
	415 + 900	Agral (0.1%v/v)

The red cotyledon lentil cultivar CDC Maxim (40–45 mg seed weight) was chosen for the study because it is the most widely grown cultivar in Canada. It is tolerant to imidazolinone herbicides. Details of field management are documented by Zhang et al. (2016). Prior to seeding, lentil seeds were inoculated with liquid Nodulator^@^ inoculant (*Rhizobium leguminosarum biovar viceae* at a rate of 2.76 mL kg^−1^ in 2012, and with Tag Team^@^ Granular (*Rhizobium leguminosarum and penicillium bilaii*) at a rate of 2.8 kg ha^−1^ in 2013. Seeds were treated with Apron Maxx RTA (0.73% fludioxonil; 1.10% metalaxyl-M and S-isomer) at a rate of 325 ml per 100 kg of seed before sowing in each site. After treatment, seeds were sown at 3 cm depth with seeding density of 130 seeds m^−2^ using a small plot drill equipped with hoe openers spaced at 22 cm between rows. Individual plots were 2.25 m wide and 6 m long at Saskatoon and 2 m wide and 5 m long at Scott, and consisted of six rows at both sites. Sowing was performed in mid-May in each year at each location. A tank mixture of imazamox and imazethapyr (30 g a.i ha^−1^) was sprayed between 5 and 6th lentil node stage at Saskatoon and quizalofop-p-ethyl (420 g a.i. ha^−1^) was sprayed at 4th node stage of lentil for post emergence weed control. Hand weeding was carried out to maintain plots weed free. The fungicides prothioconazole (166 g a.i. ha^−1^) and boscalid (294 g a.i. ha^−1^) were applied at Saskatoon and Scott, respectively, to control foliar diseases.

### Post-harvest seed measurements

Randomly selected 250-seed samples from individual plots were collected, counted using an electronic seed counter (ESC-1 Agriculex Inc., Guelph, ON, Canada), and weighed to determine 1000-seed weight. Seed diameter and thickness were measured using round-hole and slotted-hole sieves (Hossain et al., 2010; Fedoruk et al., 2013). Seed diameter was measured by passing seed samples of approximately 100 g through a set of 10 round-hole sieves ranging from 5.8 mm (15/64″) down to 3.6 mm (9/64″) in 0.25 mm (1/64″) increments. Seed thickness was measured by passing the same sample through a set of six slotted-hole sieves from 3.1 mm (8/64″) down to 2.0 mm (5/64″) in 0.2 mm (0.5/64″) increments. All samples were shaken for 1 min on a flatbed shaker (Lab Line Instruments, Melrose Park, Illinois, USA). The seed fractions remaining in each round and slotted-hole sieve were weighed. Seed diameter and thickness for each sample were calculated using the following formulas:

$$\begin{array}{l} \text{Percentage on sieve}=\frac{weight\;of\;seed\;in\;each\;sieve\;\left(g\right)}{weight\;of\;total\;sample\;seed\;(g)}\times 100\;,\\ \text{Mean seed diameter}=[\frac{\%\;of\;seed\;weight\;on\;round\;sieve\;}{100}\;\\ \;\times \;sieve\;hole\;size\;\left(mm\right)],\\ \text{Mean seed thickness}=[\frac{\%\;of\;seed\;weight\;on\;slotted\;sieve\;}{100}.\\ \;\;\times \;sieve\;hole\;size\;\left(mm\right),]\;\\ \text{ Seed plumpness}=\frac{mean\;seed\;thickness\;}{mean\;seed\;diameter\;}. \end{array}$$

### Dehulling procedure and separation of dehulled fractions

Prior to dehulling, initial moisture content of the seeds was determined using an oven dry method (AACC, 2000). For each whole seed sample, 16 g was dried at 130°C for 20 h, and the weight difference of each sample expressed as moisture percentage.

Lentil seed samples (30 g) that remained in the round (4.47–5.16 mm) and slotted (2.38–2.58 mm) sieves were tempered to 12.5% moisture (Wang, 2005) and then dehulled using a grain testing mill (TM05, Satake Engineering Co., Hiroshima, Japan) fitted with a 36-mesh abrasive wheel rotating at 1100 rpm for 38 s, as described by Wang (2005). After dehulling, milled samples were collected in a paper envelope and then the entire milled sample was weighed and separated into different fractions. For separation, the first sample was screened on Canada standard No. 14 (1.40 mm) and No. 35 mesh (850 μm) sieves. The powder was collected and weighed. The leftover fraction in the No. 14 sieve was passed through an aspiration unit to separate the hull portions. The sample seeds remaining in the aspiration column were further sieved to separate fractions. The remaining lentil seeds without powder and hulls were passed through a No. 6 (6/64″) slotted sieve and No. 9 (9/64″) round sieve. Whole lentils remaining over the slotted sieve were considered football, lentils remaining in the round sieve were considered split, and material in the pan was considered broken seed. Any whole and split lentils with adhering hulls were separated manually into their respective adhering hulled or dehulled classes. All fractions were weighed and then expressed as a proportion of the total original milled sample weight. Dehulling efficiency was defined as the percent of un-dehulled whole and split (%) seed relative to total initial sample weight (Wang, 2005; Bruce, 2008); and milling recovery indicated as the percent of dehulled splits and football fractions to total initial sample weight. Football recovery, milling recovery, and dehulling efficiency were calculated according to the following:

$$\begin{array}{l} \text{Dehulling efficiency}\;\left(\text{DE},\;\%\right)=\left[1-\frac{(weight\;of\;undehulled\;whole\;seed\;\left(g\right)+\;undehulled\;split\;seed\;\left(g\right)}{weight\;of\;sample\;seed\;\left(g\right)}\right]\times 100,\\ \text{ Milling recovery}\;(\text{MR},\;\%)=\frac{weight\;of\;milled\;seeds\;\left(g\right)}{weight\;of\;sample\;seed\;\left(g\right)}\;\times 100\\ \text{ Football recovery}\;(\text{FR},\;\%)=\frac{weight\;of\;football\;(un-split\;intact\;seed)\;\left(g\right)}{weight\;of\;sample\;seed\;\left(g\right)}\;\times 100. \end{array}$$

### Germination and vigor tests

Seed germination tests were performed at Discovery Seed Labs Ltd. (Saskatoon, SK) using the rolled paper towel method and procedures recommended for lentil seed by the Canadian Food Inspection Agency (CFIA, 2012). Two hundred seeds from each plot in each replication were evenly spaced on two sheets of germination paper and then covered with a moistened paper. Four replication were used. The sheets of paper were rolled and placed in an upright position. The rolled paper sheets were moistened daily by adding water. The temperature was maintained at 20°C. After 7 d, the number of normal seedling and abnormal seedlings, such as number of un—germinate fresh, dormant, hard and dead seed were counted. Then percentage of normal seedling were used to expresses germination percentage.

Seedling vigor was also determined by the standard method developed by Canadian Food Inspection Agency at Discovery Seed Labs Ltd., using 200-seed samples at 5°C for 7 d. The number of normal and vigorous seedlings were counted 7 d after emergence and expressed as a percentage.

### Glyphosate residue content

The glyphosate residue data, reported by Zhang et al. (2016) using high performance liquid chromatography (HPLC) column switching and post-column derivation with fluorescence detection to determine glyphosate (at ALS Laboratories, Edmonton, AB, Canada), were used for correlation analysis among selected traits. Glyphosate residue content was analyzed using both treated and un-treated seeds. Each 250 g seed sample was collected at 7 DAA from border rows, cleaned, placed into plastic bags and kept in a freezer at −20°C until all samples were collected. Samples were sent to ALS laboratory in Edmonton, AB, Canada. Using a standardized process provided by ALS Laboratories, high performance liquid chromatography (HPLC) using column switching and post-column derivatization with fluorescence detection was employed to determine glyphosate and AMPA residue. Briefly, a mixture of 150 ml of 0.1 M hydrochloric acid and 50 ml of dichloromethane was added to ground samples. The solution was homogenized for 1 min with a polytron, and centrifuged at 5000 RPM for 10 min. The aqueous layer of this solution (100 ml) was decanted to a flask and diluted with deionized water to 350 ml, and eluted through a Chelex 100 resin column at 2 drops per second. The wall of this column was then washed with 50 ml of deionized water and 100 ml of 0.2 M hydrochloric acid. All the eluent was discarded. Following this, 7 ml of 6 M hydrochloric acid was added to the column, and the eluent was discarded. 25 ml of 6 M hydrochloric acid was added again to the column, and with the eluent collected, mixed with 11 ml of concentrated hydrochloricacid and applied to a AG1-X8 resin column to remove excess iron. After the eluent entered the AG1-X8 resin column, the column was rinsed with 10 ml of 6 M hydrochloric acid, and the eluent was concentrated on a rotary evaporator. The extract of glyphosate and AMPA was then determined with an HPLC equipped with a fluorescence detector. Differential retention time was used to distinguish between glyphosate and AMPA, with a limit of detection of 0.020 ppm for both compounds.

### Data analyses

Normality and homogeneity tests were performed using residual data through the PROC UNIVARIATE procedure and Levene's test, respectively, prior to using a mixed model in SAS 9.3 (SAS Institute, Inc., Cary, NC, USA; SAS, 2015). Data were pooled and analyzed using the PROC MIXED procedure in a Randomized complete block design. The pre-harvest aid (desiccant) treatment was considered a fixed factor, whereas environment (year × location site year), environment × treatment interaction, and blocks were considered random factors. In the mixed model analysis, the significance of the fixed effect was tested using *F*-tests, whereas random effects were tested using a *Z*-test of the variance estimate. The REPEATED/GROUP statement was used to model heterogeneous variance for germination data from 2012 samples because these data did not meet the assumption to use ANOVA even after transformation. The covariance parameter estimation (COVTEST option of PROC MIXED) was used to determine whether or not data might be combined across site-years for analysis. The data were analyzed for each year and location separately for those variables that had significant interactions of site-year with desiccants. Fisher's least significant difference (LSD) was performed for mean separation with a 5% significance level. Additionally, all letter groupings for significance differences were established using PDMIX 800 in SAS (Saxton, 1998). Simple linear contrast estimate was used to compare differences in mean of groups. PROC CORR command in SAS 9.3 was used to analyze correlation among selected traits.

## Results

### Seed physical characteristics: diameter, thickness, and plumpness

Desiccation treatments had a marginal effect on 1000-seed weight and seed moisture content prior to conditioning; therefore, data for these parameters are not presented. And weather information related mean monthly temperature and precipitation during the growing season at each location in 2012 and 2013 are presented in Table 2. Irrespective of year or location, glyphosate applied alone or as a tank mix with other herbicides had no significant effect on seed diameter, thickness, or seed plumpness (Table 3). Effects on seed physical dimensions were consistent across all site-years, as no significant interactions were observed between desiccant and site-year (Table 3). However, contrast analysis showed that the addition of contact herbicides to glyphosate increased seed diameter compared to glyphosate applied alone (Table 4). Conversely, application of higher rates of contact herbicides significantly decreased seed diameter by 2%. In contrast, neither addition of contact herbicide with glyphosate nor glyphosate applied alone affected seed thickness or plumpness (Table 4). These results suggest that none of the contact herbicides considered, applied alone or in tank mixes with glyphosate, had any adverse effect on seed physical qualities.

**Table 2:** **Mean monthly temperature and total monthly precipitation during the growing season at Saskatoon and Scott, Saskatchewan, Canada, in 2012 and 2013**.

**Month**	**2012**		**2013**	
	**Mean monthly temperature (°C)**	**Total precipitation (mm)**	**Mean monthly temperature (°C)**	**Total precipitation (mm)**
***SASKATOON, SASKATCHEWAN***				
May	16.4	108.0	13.0	15.2
June	21.5	121.1	15.2	115.9
July	19.7	80.9	17.4	35.2
Aug.	17.3	48.5	18.9	14.7
Sep.	14.0	0.8	15.2	14.9
***SCOTT, SASKATCHEWAN***				
May	16.4	50.6	19.9	38.9
June	21.2	164.6	20.0	113.5
July	24.3	56.4	22.7	26.1
Aug.	23.6	51.4	24.5	63.3
Sep.	20.2	24.4	22.1	0.0

*Source: http://climate.weatheroffice.gc.ca*.

**Table 3:** ***P*-values derived from combined analysis of variance using a mixed model for seed diameter, seed plumpness, seed thickness, seed germination, seed vigor, dehulling efficiency (DE), milling recovery (MR), and football recovery (FR) influenced by desiccation treatment at Saskatoon and Scott, Saskatchewan (SK) in 2012 and 2013**.

**Source**	**Seed diameter (d)**	**Seed thickness (b)**	**Seed plumpness (b/d)**	**Seed germination**	**Seed vigor**	**DE (%)**	**MR (%)**	**FR (%)**
Site-year (SY)	0.180	0.163	0.160	0.442	0.190	0.167	0.218	0.016^*^
Block (Site-year)	0.010^*^	0.012^*^	0.013^*^	0.490	0.004^**^	0.088	0.044^*^	0.110
Desiccant (D)	0.152	0.450	0.445	<0.0001^***^	<0.0001^***^	0.152	0.294	0.028^*^
SY × D	0.110	0.070	0.058	<0.0001^***^	<0.0001^***^	0.050^*^	0.037^*^	0.050^*^

*, **, and *** *represent significant differences at P < 0.05, P < 0.01, and P < 0.001, respectively*.

**Table 4:** **Mean seed thickness, seed diameter, and seed plumpness of lentil treated by desiccant treatments at Saskatoon and Scott, SK in 2012 and 2013**.

**Desiccant treatment**	**Rate(g ai/ae ha^−1^)**	**Seed diameter (mm) (d)**	**Seed thickness (mm) (b)**	**Seed plumpness (d/b)**
Untreated control	-	4.70	2.71	0.57
Glyphosate	900	4.68	2.69	0.57
Pyraflufen-ethyl	10	4.67	2.66	0.57
Pyraflufen-ethyl	20	4.68	2.62	0.56
Glufosinate	300	4.65	2.65	0.57
Glufosinate	600	4.66	2.67	0.57
Flumioxazin	105	4.67	2.68	0.57
Flumioxazin	210	4.68	2.76	0.59
Saflufenacil	36	4.66	2.67	0.57
Saflufenacil	50	4.69	2.68	0.57
Diquat	208	4.64	2.63	0.56
Diquat	415	4.66	2.62	0.56
Pyraflufen-ethyl + glyphosate	10 + 900	4.69	2.59	0.55
Pyraflufen-ethyl + glyphosate	20 + 900	4.68	2.64	0.56
Glufosinate + glyphosate	300 + 900	4.67	2.60	0.56
Glufosinate + glyphosate	600 + 900	4.64	2.68	0.57
Flumioxazin + glyphosate	105 + 900	4.66	2.69	0.58
Flumioxazin + glyphosate	210 + 900	4.67	2.63	0.56
Saflufenacil + glyphosate	36 + 900	4.66	2.68	0.57
Saflufenacil + glyphosate	50 + 900	4.68	2.62	0.56
Diquat + glyphosate	208 + 900	4.68	2.73	0.57
Diquat + glyphosate	415 + 900	4.65	2.67	0.57
LSD		ns	ns	ns
**CONTRASTS**				
Untreated control vs. ${\text{TM}}^{\bar{\text{T}}}$ and glyphosate		0.03^*^	0.01	0.05
Glyphosate vs. ${\text{TM}}^{\bar{\text{T}}}$ +glyphosate		−0.03^*^	0.01	0.06
${\text{TM}}^{\bar{\text{T}}}$ vs. ${\text{TM}}^{\bar{\text{T}}}$ +glyphosate		0.00	0.00	−0.02
${\text{TM}}^{\bar{\text{T}}}$ (low rate) vs. ${\text{TM}}^{\bar{\text{T}}}$ (high rate)		0.02^**^	0.00	0.00

*Contrast statements indicate differences in means between treatments*.

${\text{TM}}^{\bar{\text{T}}}$, herbicides used as tank-mix; ns denotes non-significant at P < 0.05.

* and ** *represent significant differences at P < 0.05 and P < 0.01, respectively. LSD denotes Fisher's least significant differences value at 0.05 level of probability*.

**Table 5:** ***F*-values from analysis of variance (ANOVA) for seed germination, seed vigor, dehulling efficiency, milling recovery, and football recovery evaluated at Saskatoon and Scott, SK in 2012 and 2013**.

**Year**	**Location**	**Sources of variation**	**Df**	***F*****-values**				
				**Seed germination**	**Seed Vigor**	**Dehulling efficiency (%)**	**Milling recovery (%)**	**Football recovery (%)**
2012	Saskatoon	Desiccants	21	28.02^**^	4.07^***^	1.97^*^	1.82^*^	1.73^*^
	Scott	Desiccants	21	29.2^***^	7.68^***^	2.14^**^	1.84^*^	0.82^ns^
2013	Saskatoon	Desiccants	21	1.22^ns^	0.78^ns^	1.23^ns^	1.09^ns^	3.47^**^
	Scott	Desiccants	21	1.31^ns^	1.10^ns^	0.92^ns^	1.45^ns^	1.91^*^

*, **, and *** represent significant differences at P < 0.05, P < 0.01, and P < 0.001, respectively.

ns *represents non-significant. Df denotes degree of freedom*.

### Seed biological characteristics: germination and seedling vigor

The impact of desiccant treatment on seed germination and seedling vigor varied with growing environment (Table 3) so these data were analyzed separately by site year. At Saskatoon in 2012, diquat, pyraflufen, and glufosinate (300 g a.i. ha^−1^), and flumioxazin did not affect lentil seed germination compared to the untreated control; in contrast, plots sprayed with glyphosate (900 g a.i. ha^−1^) alone or with these desiccants as tank-mix with glyphosate except (diquat + glyphosate) resulted in a significant reduction of seed germination compared to the untreated control (Table 6). Furthermore, adding other contact herbicides to glyphosate significantly increased seed germination (10.9%) over glyphosate applied alone as did application of lower rates of contact herbicide alone. Similar results were observed at Scott in 2012, where seeds from plots sprayed with glyphosate alone and in combination with pyraflufen (10 or 20 g a.i. ha^−1^), glufosinate (300 g a.i. ha^−1^), flumioxazin (105 or 210 g a.i. ha^−1^), or saflufenacil (36 g a.i. ha^−1^) had significantly reduced germination compared to the untreated control (Table 6). Adding glyphosate to contact herbicides as a tank mix significantly improved seed germination (9.9%) compared to glyphosate applied alone. In 2013, no adverse effect on seed germination was attributable to glyphosate treatment at either site (Table 6).

**Table 6:** **Means comparison of seed germination (%) of lentil influenced by desiccants at Saskatoon and Scott, SK in 2012 and 2013**.

**Treatment**		**Seed germination (%)**			
	**Rate**	**Saskatoon**		**Scott**	
	**(g ai/ae ha^−1^)**	**2012**	**2013**	**2012**	**2013**
Untreated control	-	90.0^cd^	95.3	89.5^a−d^	90.7
Glyphosate	900	66.3^hi^	87.5	69.3^l^	91.0
Pyraflufen-ethyl	10	92.3^a−c^	94.8	91.3^a−c^	89.5
Pyraflufen-ethyl	20	92.0^bc^	94.3	90.3^a−c^	92.2
Glufosinate	300	86.0^a−f^	92.0	79.3^g−j^	88.8
Glufosinate	600	75.8^g^	90.8	78.5^h−j^	89.7
Flumioxazin	105	95.3^a^	96.8	91.3^a−c^	92.5
Flumioxazin	210	92.8^a−c^	94.5	93.5^a^	87.5
Saflufenacil	36	87.0^de^	95.5	88.0^a−f^	91.7
Saflufenacil	50	87.5^de^	95.3	88.8^a−e^	87.2
Diquat	208	91.5^a−e^	95.5	94.0^a^	90.0
Diquat	415	92.5^b^	94.0	93.0^a−d^	90.5
Pyraflufen-ethyl + glyphosate	10 + 900	65.3^hi^	93.0	71.0^g−l^	90.5
Pyraflufen-ethyl + glyphosate	20 + 900	71.3^g−i^	93.8	77.5^f−i^	90.2
Glufosinate + glyphosate	300 + 900	73.5^gh^	95.8	78.5^e−j^	89.5
Glufosinate + glyphosate	600 + 900	73.8^g−i^	94.5	80.0^c−i^	88.5
Flumioxazin + glyphosate	105 + 900	64.0^i^	91.3	69.3^i^	90.7
Flumioxazin + glyphosate	210 + 900	66.8^hi^	94.8	73.5^g−i^	90.7
Saflufenacil + glyphosate	36 + 900	74.5^f−i^	92.5	72.3^hi^	91.5
Saflufenacil + glyphosate	50 + 900	78.8^fg^	92.8	76.3^e−l^	89.2
Diquat + glyphosate	208 + 900	86.8^d−f^	96.0	83.8^a−g^	84.7
Diquat + glyphosate	415 + 900	86.5^d−f^	95.8	82.5^b−h^	89.5
LSD		6.70	*ns*	7.85	ns
**CONTRASTS**					
Untreated control vs. ${\text{TM}}^{\bar{\text{T}}}$ and glyphosate		12.25^***^	2.14	10.90^***^	0.45
Glyphosate vs. ${\text{TM}}^{\bar{\text{T}}}$ + glyphosate		10.87^***^	1.95	9.97^***^	0.50
${\text{TM}}^{\bar{\text{T}}}$ vs. ${\text{TM}}^{\bar{\text{T}}}$ + glyphosate		−2.88^***^	−1.33	−1.95	0.48
${\text{TM}}^{\bar{\text{T}}}$ (low rate) vs. ${\text{TM}}^{\bar{\text{T}}}$ (high rate)		5.10^**^	−0.40	4.05^*^	1.45

Contrast statements indicate differences in mean between desiccant treatments. Means followed by the same letter within a column are not significantly different (p < 0.05). ${\text{TM}}^{\bar{\text{T}}}$ represents herbicides used as tank-mix; ns denotes non-significant at P < 0.05.

*, **, and *** *represent significant differences at P < 0.05, P < 0.01, and P < 0.001, respectively. LSD denotes Fisher's least significant differences value at 0.05 level of probability*.

Similar to seed germination results, glyphosate sprayed alone or tank mix with other contact herbicides, except pyraflufen ethyl (20 g a.i. ha^−1^) and diquat (208 or 415 g a.i. ha^−1^) plus glyphosate significantly reduced seedling vigor compared to the untreated control at Saskatoon in 2012 (Table 7). On average, the addition of glyphosate to other desiccants as a tank mixture partner significantly reduced seedling vigor compared to their sole application (Table 7). Likewise, the glyphosate, glufosinate (600 g a.i. ha^−1^), saflufenacil (36 or 50 g a.i. ha^−1^), pyraflufen (100 g a.i. ha^−1^ or20 g a.i. ha^−1^) + glyphosate, glufosinate (300 g a.i. ha^−1^) + glyphosate (900 g a.e. ha^−1^), flumioxazin (105 or 210 g a.i. ha^−1^)^1^+ glyphosate, and saflufenacil (36 or 50 g a.i. ha^−1^) + glyphosate treatments resulted in a significant reduction of seedling vigor compared to the control at Scott in 2012. Overall, the addition of glyphosate to other desiccants as a tank mix significantly reduced seed vigor compared to desiccants applied alone. The high rates of sole application of tank mix herbicides also significantly reduced seedling vigor compared to the lower rate of these herbicides applied alone (Table 7).

**Table 7:** **Means comparison of seed vigor (%) of lentil influenced by desiccants at Saskatoon and Scott, SK in 2012 and 2013**.

**Treatment**	**Rate**	**Seed vigor (%)**			
		**Saskatoon**		**Scott**	
	**(g ai/ae ha^−1^)**	**2012**	**2013**	**2012**	**2013**
Untreated control	-	78.0^a−c^	89.5	87.7^a−c^	83.0
Glyphosate	900	65.0^d−h^	87.5	68.7^hi^	82.2
Pyraflufen-ethyl	10	75.3^a−d^	86.7	82.2^b−e^	85.0
Pyraflufen-ethyl	20	75.3^a−d^	89.5	86.2^a−d^	82.0
Glufosinate	300	78.5^ab^	80.5	80^c−f^	82.0
Glufosinate	600	70.0^b−f^	84.2	72.7^f−i^	81.8
Flumioxazin	105	86.3^a^	90.7	87.0^a−c^	82.7
Flumioxazin	210	76.0^a−d^	91.5	89.5^ab^	80.7
Saflufenacil	36	70.3^b−e^	90.0	73.0^f−i^	78.0
Saflufenacil	50	61.8^e−h^	87.2	76.5^e−h^	84.7
Diquat	208	77.8^a−c^	89.6	91.0^a^	84.7
Diquat	415	78.3^a−c^	88.0	89.0^ab^	85.0
Pyraflufen-ethyl + glyphosate	10 + 900	61.5^e−h^	86.8	74.7^e−i^	85.3
Pyraflufen-ethyl + glyphosate	20 + 900	67.0^c−g^	86.5	69.0^hi^	78.7
Glufosinate + glyphosate	300 + 900	66.2.4^a−d^	88.5	71.7^g−i^	81.3
Glufosinate + glyphosate	600 + 900	66.3^d−h^	88.7	75.2^e−i^	82.5
Flumioxazin + glyphosate	105 + 900	55.5^h^	86.5	69.5^hi^	84.5
Flumioxazin + glyphosate	210 + 900	57.3^gh^	83.5	68.5^i^	80.5
Saflufenacil + glyphosate	36 + 900	65.0^d−h^	86.5	70.5^hi^	76.5
Saflufenacil + glyphosate	50 + 900	58.8^f−h^	86.0	72.7^f−i^	82.0
Diquat + glyphosate	208 + 900	72.5^b−e^	83.5	82.5^b−e^	82.5
Diquat + glyphosate	415 + 900	71.8^b−e^	87.5	78.7^d−g^	81.7
LSD		11.37	*ns*	7.81	ns
**CONTRASTS**					
Untreated control vs. ${\text{TM}}^{\bar{\text{T}}}$ and glyphosate		8.30^**^	1.98	11.63^***^	0.45
Glyphosate vs. ${\text{TM}}^{\bar{\text{T}}}$ + glyphosate		6.88^*^	1.88	10.97^***^	0.65
${\text{TM}}^{\bar{\text{T}}}$ vs. ${\text{TM}}^{\bar{\text{T}}}$ + glyphosate		2.20	0.13	−1.22	0.67
${\text{TM}}^{\bar{\text{T}}}$ (low rate) vs. ${\text{TM}}^{\bar{\text{T}}}$ (high rate)		1.40	0.90	6.20^***^	2.25

Contrast statements indicate differences in means between lentil desiccant treatments. Means followed by the same letter within a column are not significantly different (p < 0.05). ${\text{TM}}^{\bar{\text{T}}}$ represents herbicides used as tank-mix; ns denotes non-significant at P < 0.05.

*, **, and *** *represent significant differences at P < 0.05, P < 0.01, and P < 0.001, respectively. LSD denotes Fisher's least significant differences value at 0.05 level of probability*.

Conversely, none of the desiccant treatments had a significant effect on seedling vigor in 2013 (Table 7). The lack of adverse effects and differences of treatments may have resulted from reduction of glyphosate translocation to lentil seed during desiccation and lower seed moisture content at the time of treatment application. Seed moisture at the time of application was 32 and 35% at Saskatoon and Scott in 2013, respectively, compared to 35 and 40% in 2012.

### Milling characteristics: dehulling efficiency, milling recovery, and football recovery

#### Dehulling efficiency (%)

The desiccant by site-year interaction was significant for dehulling efficiency, milling recovery, and football recovery (Table 3), and thus these data were analyzed within site-years (Table 5). At Saskatoon in 2012, only application of pyraflufen (20 g a.i. ha^−1^) with glyphosate (900 g a.e. ha^−1^) significantly reduced dehulling efficiency compared to the control. Application of diquat (415 g a.i. ha^−1^) increased dehulling efficiency by 5.6% over pyraflufen (20 g a.i. ha^−1^) with glyphosate (Table 8). The contrast results show application of glyphosate in tank mixtures significantly lowered dehulling efficiency compared to sole application of all contact herbicides.

**Table 8:** **Means comparison of dehulling efficiency (%) of lentil treated with desiccants at Saskatoon and Scott, SK in 2012 and 2013**.

**Treatment**	**Rate (g ai/ae ha-1)**	**Dehulling efficiency (%)**			
		**Saskatoon**		**Scott**	
		**2012**	**2013**	**2012**	**2013**
Untreated control	-	92.9^a−d^	97.5	94.1^a−c^	96.7
Glyphosate	900	92.3^a−e^	96.3	94.2^a−c^	96.5
Pyraflufen-ethyl	10	93.0^a−d^	97.7	93.7^bc^	95.1
Pyraflufen-ethyl	20	91.8^a−e^	98.2	93.6^bc^	95.3
Glufosinate	300	92.9^a−e^	97.6	93.7^bc^	95.1
Glufosinate	600	92.2^a−e^	97.6	94.8^ab^	96.4
Flumioxazin	105	93.3^a−c^	97.9	93.8^a−c^	96.2
Flumioxazin	210	92.2^a−e^	97.7	94.1^a−c^	96.4
Saflufenacil	36	92.1^a−e^	97.6	91.1^d^	94.7
Saflufenacil	50	90.4^d−f^	98.1	92.6^cd^	96.5
Diquat	208	93.0^a−c^	97.9	95.0^ab^	95.6
Diquat	415	94.1^a^	97.8	95.4^a^	95.6
Pyraflufen-ethyl + glyphosate	10 + 900	90.7^c−f^	97.1	94.2^a−c^	95.6
Pyraflufen-ethyl + glyphosate	20 + 900	88.5^f^	97.1	94.4^ab^	96.6
Glufosinate + glyphosate	300 + 900	90.3^ef^	97.5	94.5^ab^	96.4
Glufosinate + glyphosate	600 + 900	91.2^b−e^	98.0	94.4^ab^	96.1
Flumioxazin + glyphosate	105 + 900	92.8^a−e^	97.9	93.4^bc^	96.6
Flumioxazin + glyphosate	210 + 900	92.9^a−d^	97.9	93.9^a−c^	96.1
Saflufenacil + glyphosate	36 + 900	91.6^a−e^	97.5	93.7^a−c^	95.2
Saflufenacil + glyphosate	50 + 900	92.8^a−e^	98.1	94.1^a−c^	96.2
Diquat + glyphosate	208 + 900	93.7^ab^	97.5	95.0^ab^	96.2
Diquat + glyphosate	415 + 900	92.4^a−e^	98.2	94.5^ab^	96.0
LSD		2.62	*ns*	1.70	*ns*
**CONTRASTS**					
Untreated control vs. ${\text{TM}}^{\bar{\text{T}}}$ and glyphosate		1.36	−0.07	0.06	0.71
Glyphosate vs. ${\text{TM}}^{\bar{\text{T}}}$ + glyphosate		1.48	−0.03	−0.01	0.77
${\text{TM}}^{\bar{\text{T}}}$ vs. ${\text{TM}}^{\bar{\text{T}}}$ + glyphosate		−1.08^*^	−0.37	0.48	0.02
${\text{TM}}^{\bar{\text{T}}}$ (low rate) vs. ${\text{TM}}^{\bar{\text{T}}}$ (high rate)		1.49	−0.21	−0.36	−0.24

Contrast statements indicate differences in mean between desiccant treatments in lentil. Means followed by the same letter within a column are not significantly different (p < 0.05). TM represents herbicides used as tank-mix; ns denotes non-significant at P < 0.05.

* *Represents significant differences at P < 0.05, LSD denotes Fisher's least significant differences value at 0.05 level of probability*.

At Scott in 2012, most desiccant treatments exhibited better or comparable dehulling efficiencies (%) compared to the untreated control; the only exception was treatment with saflufenacil (36 g a.i. ha^−1^), which led to significantly reduced dehulling efficiency. Application of the high rate of diquat (415 g a.i. ha^−1^) increased dehulling efficiency by 4.3% over application of saflufenacil (36 g a.i. ha^−1^) alone. None of treatments applied at either rate had a significant impact on dehulling efficiency percentages at either Saskatoon or Scott in 2013 (Table 8).

#### Milling recovery (%)

At Saskatoon in 2012, most desiccant treatments had no significant effect on milling recovery. The glufosinate (300 g a.i. ha^−1^) and Pyraflufen (20 g a.i. ha^−1^) glyphosate (900 g a.e. ha^−1^) treatment significantly reduced milling recovery compared to control. While diquat (415 g a.i. ha^−1^) increased milling recovery by 5.0% compared to the Pyraflufen (20 g a.i. ha^−1^) with glyphosate treatment (Table 9). On average, adding glyphosate to desiccants reduced milling recovery by 1.2% compared to sole application of glyphosate. The low application rate of other contact herbicides increased milling recovery (1.4%) compared to the corresponding high rates.

**Table 9:** **Means comparison of milling recovery (%) of lentil treated with desiccants at Saskatoon and Scott, SK in 2012 and 2013**.

**Treatment**	**Rate**	**Milling Recovery (%)**			
		**Saskatoon**		**Scott**	
	**(g ai/ae ha^−1^)**	**2012**	**2013**	**2012**	**2013**
Untreated control	-	81.0^a−d^	83.6	79.3^ab^	84.9
Glyphosate	900	80.2^a−e^	82.9	79.4^ab^	84.3
Pyraflufen-ethyl	10	80.7^a−e^	83.8	78.9^ab^	83.9
Pyraflufen-ethyl	20	79.7^a−f^	84.2	79.1^ab^	83.2
Glufosinate	300	78.2^ef^	83.3	79.2^ab^	83.6
Glufosinate	600	80.6^a−e^	83.7	79.6^a^	84.5
Flumioxazin	105	81.3^a−c^	84.0	79.5^ab^	84.0
Flumioxazin	210	80.5^a−e^	83.7	79.2^ab^	82.3
Saflufenacil	36	79.7^a−e^	83.2	76.2^b^	82.8
Saflufenacil	50	79.5^b−f^	83.6	78.4^ab^	84.3
Diquat	208	80.9^a−d^	84.5	80.1^*a*^	83.5
Diquat	415	82.2^a^	83.6	80.8^a^	83.2
Pyraflufen-ethyl + glyphosate	10 + 900	78.8^c−f^	82.9	78.7^ab^	83.8
Pyraflufen-ethyl + glyphosate	20 + 900	77.2^f^	83.4	79.1^ab^	85.3
Glufosinate + glyphosate	300 + 900	78.5^d−f^	82.5	79.5^ab^	84.8
Glufosinate + glyphosate	600 + 900	79.3^b−f^	83.5	79.4^ab^	84.2
Flumioxazin + glyphosate	105 + 900	80.8^a−e^	83.9	78.4^ab^	86.0
Flumioxazin + glyphosate	210 + 900	81.0^a−d^	83.4	79.4^ab^	84.0
Saflufenacil + glyphosate	36 + 900	79.6^b−f^	83.7	78.7^ab^	82.9
Saflufenacil + glyphosate	50 + 900	80.4^a−e^	83.3	79.2^ab^	83.8
Diquat + glyphosate	208 + 900	81.8^a^	83.2	79.8^a^	83.8
Diquat + glyphosate	415 + 900	78.9^c−f^	83.7	79.8^a^	83.7
LSD		2.58	ns	1.76	ns
**CONTRASTS**					
Untreated control vs. ${\text{TM}}^{\bar{\text{T}}}$ and glyphosate		1.43	0.41	0.09	0.57
Glyphosate vs. ${\text{TM}}^{\bar{\text{T}}}$ + glyphosate		1.55	0.49	0.01	0.73
${\text{TM}}^{\bar{\text{T}}}$ vs. ${\text{TM}}^{\bar{\text{T}}}$ + glyphosate		−1.19^**^	−0.42	0.34	0.49
${\text{TM}}^{\bar{\text{T}}}$ (low rate) vs. ${\text{TM}}^{\bar{\text{T}}}$ (high rate)		1.35^*^	0.15	−0.32	−0.43

Contrast statements indicate differences in mean between desiccant treatments. Means followed by the same letter within a column are not significantly different (p < 0.05). ${\text{TM}}^{\bar{\text{T}}}$ represents herbicides used as tank-mix; ns denotes non-significant at P < 0.05.

* and ** *represents significant differences at P < 0.05 and P < 0.01, respectively, LSD denotes Fisher's least significant differences value at 0.05 level of probability*.

At Scott in 2012, only plots treated with saflufenacil (36 g a.i. ha^−1^) had significantly lower milling recovery yield compared to the diquat treated plots. Application of the high rate of diquat (415 g a.i. ha^−1^) increased milling recovery by 4.6% over the low rate of saflufenacil application. Contrast comparisons showed that neither sole application nor herbicide mixes with glyphosate significantly affected milling recovery. No differences in milling recovery were observed between high and low rates of herbicide application treatments (Table 9).

No desiccant treatments affected milling recovery (%) of lentil at Saskatoon or Scott in 2013 (Table 9). Milling recovery was also unaffected by either the addition of contact herbicides to glyphosate or application rate of these herbicides.

#### Football recovery (%)

Football recovery yield was influenced by growing environment. At Saskatoon in 2012, most desiccant treatments had a marginal effect on football recovery (Table 10); the exception was significantly reduced recovery for the saflufenacil (50 g a.i. ha^−1^) + glyphosate (900 g a.e. ha^−1^) treatment compared to the control. Application of diquat (207 g a.i. ha^−1^) increased football recovery by 9.5% compared to saflufenacil (50 g a.i. ha^−1^) + glyphosate (900 g a.e. ha^−1^). On average, adding glyphosate to other desiccants did not reduce football recovery compared to their sole application at either application rate. At Saskatoon in 2013, application of flumioxazin (210 g a.i. ha^−1^), diquat (207 g a.i. ha^−1^), and saflufenacil (36 g a.i. ha^−1^) with glyphosate significantly improved football recovery compared to the untreated control. At Scott in 2012, none of desiccants applied alone or as a tank mixture with glyphosate significantly reduced football recovery compared to the control. At Scott in 2013, only application of glufosinate (300 g a.i. ha^−1^) with glyphosate and saflufenacil (50 g a.i. ha^−1^) with glyphosate significantly decreased the football recovery compared to the untreated control. Overall, glyphosate tank mixes with other contact herbicides or these herbicides applied in higher doses had no significant impact on football recovery in any site year (Table 10).

**Table 10:** **Means comparison of football recovery (%) of lentil influenced by application of desiccants at Saskatoon and Scott, SK in 2012 and 2013**.

**Treatment**	**Rate (g ai/ae ha^−1^)**	**Football recovery (%)**			
		**Saskatoon**		**Scott**	
		**2012**	**2013**	**2012**	**2013**
Untreated control	-	48.1^a−d^	10.1^c−e^	13.7	20.1^a−c^
Glyphosate	900	46.5^b−e^	9.7^c−e^	12.2	17.9^a−e^
Pyraflufen-ethyl	10	49.9^ab^	10.9^c−e^	12.4	20.6^ab^
Pyraflufen-ethyl	20	47.2^b−e^	11.5^b−d^	14.6	16.9^b−f^
Glufosinate	300	49.9^ab^	10.9^c−e^	12.7	21.4^a^
Glufosinate	600	45.5^b−e^	10.3^c−e^	14.0	16.4^c−f^
Flumioxazin	105	47.8^a−d^	10.4^c−e^	12.2	18.4^a−d^
Flumioxazin	210	46.9^b−e^	15.1^a^	12.8	18.0^a−e^
Saflufenacil	36	43.9^de^	9.2^de^	11.6	18.0^a−e^
Saflufenacil	50	47.2^b−e^	9.6^c−e^	12.7	16.8^b−f^
Diquat	208	52.2^a^	13.4^ab^	12.3	16.6^c−f^
Diquat	415	50.1^ab^	11.6^bc^	15.0	16.2^d−f^
Pyraflufen-ethyl + glyphosate	10 + 900	45.5^b−e^	9.3^c−e^	11.8	18.7^ad^
Pyraflufen-ethyl + glyphosate	20 + 900	44.9^c−e^	10.8^c−e^	12.0	17.9^a−e^
Glufosinate + glyphosate	300 + 900	49.6^a−c^	9.0^e^	12.4	13.4^f^
Glufosinate + glyphosate	600 + 900	48.5^a−d^	10.1^c−e^	12.5	18.9^a−d^
Flumioxazin + glyphosate	105 + 900	48.4^a−d^	9.4^c−e^	12.3	17.4^b−e^
Flumioxazin + glyphosate	210 + 900	47.6^a−d^	10.3^c−e^	15.0	19.6^a−d^
Saflufenacil + glyphosate	36 + 900	47.4^b−e^	13.4^ab^	13.6	19.1^a−d^
Saflufenacil + glyphosate	50 + 900	42.7^e^	9.7^c−e^	11.2	14.5^ef^
Diquat + glyphosate	208 + 900	48.6^a−c^	9.5^c−e^	12.0	16.6^c−f^
Diquat + glyphosate	415 + 900	47.2^b−e^	11.7^bc^	14.4	17.5^b−e^
LSD		4.70	2.36	ns	3.79
**CONTRASTS**					
Untreated control vs. ${\text{TM}}^{\bar{\text{T}}}$ and glyphosate		0.53	−0.08	0.98	2.11
Glyphosate vs. ${\text{TM}}^{\bar{\text{T}}}$ + glyphosate		0.62	0.16	1.04	2.06
${\text{TM}}^{\bar{\text{T}}}$ vs. ${\text{TM}}^{\bar{\text{T}}}$ + glyphosate		0.30	−0.74	0.06	0.72
${\text{TM}}^{\bar{\text{T}}}$ (low rate) vs. ${\text{TM}}^{\bar{\text{T}}}$ (high rate)		−0.22	0.09	0.26	1.21

*Contrast statements indicate mean differences between desiccant treatments in lentil. Means followed by the same letter within a column are not significantly different (p < 0.05). ${\text{TM}}^{\bar{\text{T}}}$ represents herbicides used as tank-mix; ns denotes non-significant at P < 0.05. LSD denotes Fisher's least significant differences value at 0.05 level of probability*.

### Correlation among lentil seed morphology traits, glyphosate residue, and milling characteristics

Pearson correlation coefficients (r) were calculated for glyphosate residue content in seeds with other parameters measured in this study. Data were averaged for three replications and combined over both site-years (Saskatoon and Scott) in 2012 and 2013 for correlation analysis. Positive and high correlation was observed between seed thickness and seed plumpness (*r* = 0.97, *p* < 0.001), seed diameter (*r* = 0.62, *p* < 0.001), and glyphosate residue content (*r* = 0.51, *p* < 0.001). Seed thickness was negatively correlated with seed germination, (*r* = −0.46, *p* < 0.001), seedling vigor (*r* = −0.26, *p* < 0.01), dehulling efficiency (*r* = −0.33, *p* < 0.01), and milling recovery (*r* = −0.63, *p* < 0.001; Table 11). Seed diameter was positively and significantly correlated with seed plumpness (*r* = 0.51, *p* < 0.001) and glyphosate residue content (*r* = 0.53, *p* < 0.001) and negatively correlated with other biological traits (Table 11). Likewise, seed plumpness was only positively correlated with glyphosate residue content (*r* = 0.48, *p* < 0.001). Seed germination (*r* = −0.84, *p* < 0.001) and seedling vigor (*r* = −0.62, *p* < 0.001) were negatively and significantly correlated with glyphosate residues. Percent seed germination was positively correlated with seed vigor (*r* = 0.75, *p* < 0.001), dehulling efficiency (*r* = 0.68, *p* < 0.001), and milling recovery (*r* = −0.62, *p* < 0.001).

**Table 11:** **Correlation coefficients among lentil seed morphology traits, glyphosate residue, and milling characteristics of lentil**.

**Traits**	**STH**	**SD**	**SP**	**SG**	**SV**	**DE**	**FR**	**MR**	**GR**
STH	-								
SD	0.62^***^	−							
SP	0.97^***^	0.51^***^	-						
SG	−0.46^***^	−0.46^***^	−0.42^***^	-					
SV	−0.26^**^	−0.39^***^	−0.23^*^	0.75^***^	-				
DE	−0.33^**^	−0.45^***^	−0.29^***^	0.68^***^	0.75^***^	-			
FR	−0.16^*ns*^	0.31^***^	−0.21^*^	−0.49^***^	0.68^***^	−0.64^***^	-		
MR	−0.63^***^	−0.68^***^	−0.57^***^	0.62^***^	0.49^***^	0.76^***^	−0.23^*^	-	
GR	0.51^***^	0.53^***^	0.48^***^	−0.84^***^	−0.62^***^	−0.55^***^	0.30^**^	−0.62^***^	-

STH, Seed thickness; SD, Seed diameter; SP, Seed plumpness; SG, Seed germination; SV, Seed vigor; DE, Dehulling efficiency; FR, Football recovery; MR/, Milling recovery; GR, glyphosate residue.

*, **, and *** *indicate significance at P < 0.05, P < 0.01, and P < 0.001, respectively; ns, non-significant*.

For milling characteristics, dehulling efficiency was significantly but negatively correlated with glyphosate residue (*r* = −0.55, *p* < 0.001) and football recovery (*r* = −0.64, *p* < 0.001). Milling recovery also strongly but negatively correlated with glyphosate residues (*r* = −0.62, *p* < 0.001). In contrast, football recovery was positively correlated with glyphosate resides (*r* = 0.30, *p* < 0.01; Table 11).

## Discussion

Uniform and early seed maturity is critical to produce high quality lentil on the Canadian Prairies. Extreme growing conditions combined with the heterogeneity of soil, precipitation patterns, and the indeterminate growth habit of lentil plants often result in uneven maturation of the crop. Crop desiccants are used as pre-harvest aids to rapidly dry vegetative and reproductive plant tissues, including seeds, without affecting seed yield and quality (Ratnayake and Shaw, 1992; Soltani et al., 2013). Most lentil growers in Western Canada use herbicidal desiccants to overcome challenges of heterogeneous maturity of the crop. However, some desiccants directly impact physiological aspects of different crop species, such as mean seed weight, seed germination, and dehulling efficiency (Darwent et al., 2000; Bruce, 2008).

The current study determined the impact of the use of contact herbicides as harvest aids as applied alone or in combination with glyphosate on selected physical, physiological, and processing characteristics of lentils. None of the desiccants applied alone or in tank mixes with glyphosate adversely affected seed physical qualities, including seed diameter, thickness, and plumpness. These results are similar to Ratnayake and Shaw (1992) who report that pre-harvest use of glufosinate, glyphosate, or paraquat had no significant adverse effect on seed yield or quality in soybean when applied at the full maturity stage. Similarly, Zhang et al. (2016) and McNaughton et al. (2015) observed no reduction in yield or thousand seed weight when desiccants were applied to lentil and dry bean, respectively. Wilson and Smith (2002) report that glufosinate, paraquat, and diquat applied as desiccants to common bean accelerated seed maturity and desiccation. Glyphosate applied as a desiccant reduced pod length and seed weight when used as a harvest aid in cowpea (*Vigna unguiculata* L.) (Cedeira et al., 1985). Many studies report reduced soybean yield and seed quality as a result of the application of desiccant prior to crop maturity (Azlin and Mcwhorter, 1981; Cerkauskas et al., 1982; Boudreaux and Griffin, 2011). On the other hand, Soltani et al. (2013) observed that the addition of diquat, glufosinate, carfentrazone, flumioxazin, or saflufenacil to glyphosate improved the drying of dry bean foliage and yield. The variability among results may be related to the timing of desiccant application. The application of desiccants before physiological maturity can inhibit photosynthesis or, because lentil is an indeterminate crop where bottom pods matured before upper canopy's pods, may damage immature seeds.

In 2012, the application of glyphosate alone or as a tank mix with other herbicides (except diquat + glyphosate) significantly reduced germination percentage in lentil seeds compared to the untreated control. These results are similar to those reported by Yenish and Young (2000), who found that pre-harvest glyphosate-treated wheat had a 2–46% lower seed germination than the control. Similarly, Hampton and Hebblethwaite (1982) found that pre-harvest application of glyphosate significantly lowered ryegrass (*Lolium perenne* L.) seed germination due to production of abnormal seedlings. They also reported that germination percentage of the glyphosate-treated seeds decreased with storage. Bennett and Shaw (2000a) report that sodium chlorate plus glyphosate or paraquat applied as a pre-harvest aid to sicklepod (*Senna obtusifolia. L*) 14 days before harvest significantly reduced shoot growth and seed germination.

The reduced seed germination of glyphosate-treated plants may be caused by translocation of glyphosate to the maturing seeds and embryo, the major sink during the maturation process. Glyphosate inhibits the shikimate acid pathway for synthesis of branched aromatic amino acids, such as phenylalanine, tyrosine, and tryptophan (Vivancos et al., 2011). Tryptophan is a direct precursor of indole-3-acetic acid (IAA), which affects coleoptile elongation and shoot and root initiation (Taiz and Zeiger, 1998). Clay and Griffin (2000) suggest that use of glyphosate as a desiccant during the plant maturation phase may affect the level of IAA, the main endogenous auxin, thereby causing inhibition of germination and growth. Unlike glyphosate, diquat applied alone or with glyphosate, pyraflufen, glufosinate (300 g a.i. ha^−1^), flumioxazin, and saflufenacil applied alone did not affect seed germination in the present study. Ratnayake and Shaw (1992) report similar results. Whigham and Stoller (1979) also observed paraquat applied as a harvest aid had no effect on germination percentage of soybean. All of these are contact herbicides with limited phloem mobility, and therefore low levels of these compounds would reach the seed.

Our study showed no adverse effect of glyphosate or any contact herbicides on seed germination of lentil at either site in 2013. This might be due to lower moisture content in seeds due to reduced rainfall at the time of application (Table 2). Low rainfall hastened the dry down process of lentil crops in 2013. Zhang et al. (2016) reported glyphosate residue in lentil seeds content <2 ppm from Saskatoon and Scott when they had 32 and 35% moisture content in 2013 compared with 35 and 40% in 2012, respectively. Lower moisture content in seed harvested in 2013 might have reduced translocation of glyphosate in seeds. The application of glyphosate as a desiccant in lentil (Zhang et al., 2017) and common bean (McNaughton et al., 2015) prior to 30% seed moisture content, increases its residue to an unacceptable level (>2 ppm) causing reduced seed yield and mean seed weight. Higher translocation of glyphosate into seeds may also reduce seed germination and vigor as developing seeds are major photosynthesis sinks (Zhang et al., 2016). The significant and negative correlation between glyphosate residue and seed germination results support this explanation. Different countries do have different import policies in terms of the amount of glyphosate residue they will accept for import of lentil (Pratt, 2011). The current MRLs for glyphosate in lentil are 2 ppm, 4 ppm and 10 ppm for Canada, Japan, and the European Union (EU), respectively (Zhang et al., 2017).

A significant reduction in lentil seedling vigor was observed at Saskatoon and Scott in 2012 when glyphosate was sprayed alone or as a tank mix with other herbicides but no such differences were observed in 2013. The 2012 results are comparable to those of Hampton and Hebblethwaite (1982), who show that seedling vigor of perennial rye grass (*Lolium perenne* L.) is reduced when glyphosate is applied as a pre-harvest aid. The pre-harvest application of glyphosate is also reported to cause poor seed germination and reduced seedling vigor when applied at >40% seed moisture content in field pea and soybean (Bennett and Shaw, 2000b; Baig et al., 2003). Our study showed seedling vigor was highly negatively correlated with glyphosate residue in lentil seeds. Adverse effects of pre-harvest glyphosate application on seedling growth and vigor might be related to the reduction and physiological inactivation of mineral nutrients, such as Ca and Mn, due to glyphosate in the seeds (Cakmak et al., 2009). Mineral nutrients can play an important role in seed viability and seedling vigor and establishment, particularly under adverse soil conditions (Welch, 1999).

The growing environment can have a significant effect on dehulling efficiency of lentil (Bruce, 2008). Results from the present study show the effects of desiccants on dehulling efficiency, milling recovery, and football recovery of lentils depend to a certain extent on growing environment and moisture content in seeds at the time of application. We observed that pyraflufen (20 g a.i. ha^−1^) with glyphosate (900 g a.e. ha^−1^) and saflufenacil (36 g a.i. ha^−1^) reduced dehulling efficiency at Saskatoon and Scott in 2012, respectively. This results were concurrent with the studies on the effect of desiccation with saflufenacil in lentil (Zhang et al., 2017) and common bean (McNaughton et al., 2015) who found that dramatically reduced mean seed weight and seed yield if application of saflufenacil was made prior to 30% seed moisture content. The saflufenacil residue in the seed was reported as 0.03 mg kg^−1^. Adding saflufenacil to glyphosate did not reduced glyphosate residue in lentil compared to glyphosate applied alone, yet they found that tank mixture significantly reduced seed residue content of saflufenacil and improved crop desiccation. Saflufenacil residues present in harvested lentils may be a concern for lentil growers when it is used as a harvest aid at pre-harvest stages of crop because major lentil importing countries have also set MRLs for saflufenacil (Bryant Christie Inc, 2015).

Reduction of dehulling efficiency due to saflufenacil applied alone or as a tank mix with pyraflufen-ethyl might have occurred because these herbicides belong to the uracil and phenyl pyrazole classes, respectively, which are protoporphyrinogen oxidase (PPO) inhibitors (Grossmann et al., 2010). PPO inhibitors bind sites of Protogen IX in the chloroplast, which causes peroxidation of foliar cell membrane lipids and subsequent rapid loss of membrane integrity and necrosis (Duke et al., 1991; Grossmann et al., 2010). Both saflufenacil and pyraflufen have limited mobility in phloem (Liebl et al., 2008). These herbicides translocate mainly through the xylem, and their slow mobility may result in accumulation in seeds, thereby interfering with the normal chemical composition and alignment of the bonding layer between the lentil seed coat and the cotyledon.

Pre-harvest application of the low rate of pyraflufen with glyphosate or sole application of saflufenacil (36 g a.i. ha^−1^) resulted in reduced milling recovery at Saskatoon and Scott, respectively, in 2012. Application of diquat with or without glyphosate seemed to improve milling recovery in 2012. In contrast, Bruce (2008) reports that, during dry harvest conditions, no significant differences in milling recovery were evident between swathing and desiccation pre-harvest treatments. In our study, differential effects of the pre-harvest treatments on football recovery percentage were observed in both years but only in Saskatoon. Similar to the dehulling efficiency, saflufenacil (50 g a.i. ha^−1^) combined with glyphosate resulted in the lowest football recovery at Saskatoon in 2012. Different responses of crop harvest aids among years (2012 vs. 2013) and sites might have been the result of wet field conditions during the harvesting period and differences in moisture content and glyphosate residue in seeds. Our study shows that milling parameters, particularly dehulling efficiency and milling recovery, are inversely related to glyphosate residue in seeds. Zhang et al. (2016) report that seed moisture content in seeds at the time of application can strongly impact glyphosate translocation to seeds. They note that, irrespective of desiccation treatments, high moisture content in seeds at the time of application results in accumulation of high (>2.0 ppm) of glyphosate residues compared to lower moisture in seeds while they desiccate lentil crops. In most cases (two exceptions), however, desiccant treatments had no significant impact on football recovery at Scott in either year. These results are comparable with those of Bruce (2008), who report no differences in football recovery between swathing and desiccation with diquat when treatments were applied at a later stage of plant maturity; however, early application of diquat decreased football recovery. He suggests that desiccation by diquat caused lentil seeds to separate at the cotyledons more easily compared to swathing followed by natural drying. Swathing allows biological processes related to cotyledon binding to continue for a given period, whereas desiccation may cease the processes rapidly and therefore make the seeds more brittle.

The inconsistency of some of the results in our study in relation to seed biological qualities and milling recovery over environment indicate that further research over many environments may be required to determine the consistency of treatment effects and their economic impact. Moisture content of seeds is a key cause of translocation of glyphosate residue to seeds, which can result in adverse effects on both seed biological and milling qualities. Therefore, glyphosate is recommended for use as a desiccant in lentil once the seed moisture is 30% or less (Saskatchewan Ministry of Agriculture, 2016). Future research could focus on the relationship between moisture content of seeds and their other milling and post-harvest qualities. Environmental variation and the nature of herbicide sensitivity of lentil crops may result in different impacts on seed quality; this has been demonstrated in differences of sensitivity among crops species to flumioxazin, saflufenacil, and pyraflufen (Ivany, 2005; Soltani et al., 2010) in dry bean.

## Conclusions

Use of glyphosate alone or in tank mix with other contact herbicides as pre-harvest aids in lentil production adversely affected seed germination and seedling vigor particularly if glyphosate is translocated to the seeds. Consistent improvements in milling recovery and dehulling efficiency of lentil were only observed when diquat was used alone or in tank mix with glyphosate, suggesting that lentil growers should consider these desiccant treatments to optimize dry-down of lentil without harming seed quality if they wish to gain premium prices for red lentil based on milling efficiency.

## Author contributions

MS conducted the experiment, data collection, data analysis, interpretation, summarized the results and drafting manuscript. CW supervised the field experiments and assisted with editing the manuscript. AV Co-conceptualized and coordinated and directed the project. AV edited, revised and reviewed and drafting manuscript. All authors read and approved the final manuscript.

## Funding

This research was supported by the Agriculture Development Fund of the Saskatchewan Ministry of Agriculture, the University of Saskatchewan Department of Plant Sciences, Saskatchewan Pulse Growers and the government of Canada through the NSERC Industrial Research Chair in Lentil Genetic Improvement.

### Conflict of interest statement

The authors declare that the research was conducted in the absence of any commercial or financial relationships that could be construed as a potential conflict of interest.
